# Seasonal Variation of the Atmospheric Bacterial Community in the Greenlandic High Arctic Is Influenced by Weather Events and Local and Distant Sources

**DOI:** 10.3389/fmicb.2022.909980

**Published:** 2022-07-08

**Authors:** Lasse Z. Jensen, Marianne Glasius, Sven-Erik Gryning, Andreas Massling, Kai Finster, Tina Šantl-Temkiv

**Affiliations:** ^1^Section for Microbiology, Department of Biology, Aarhus University, Aarhus, Denmark; ^2^Arctic Research Centre, Aarhus University, Aarhus, Denmark; ^3^iCLIMATE Aarhus University Interdisciplinary Centre for Climate Change, Roskilde, Denmark; ^4^Department of Chemistry, Aarhus University, Aarhus, Denmark; ^5^DTU Wind and Energy Systems, Technical University of Denmark, Roskilde, Denmark; ^6^Department of Environmental Science, Aarhus University, Roskilde, Denmark; ^7^Department of Physics and Astronomy, Stellar Astrophysics Centre, Aarhus University, Aarhus, Denmark

**Keywords:** bioaerosols, atmospheric bacterial community, Arctic haze, microbial activity, ice nucleation

## Abstract

The Arctic is a hot spot for climate change with potentially large consequences on a global scale. Aerosols, including bioaerosols, are important players in regulating the heat balance through direct interaction with sunlight and indirectly, through inducing cloud formation. Airborne bacteria are the major bioaerosols with some species producing the most potent ice nucleating compounds known, which are implicated in the formation of ice in clouds. Little is known about the numbers and dynamics of airborne bacteria in the Arctic and even less about their seasonal variability. We collected aerosol samples and wet deposition samples in spring 2015 and summer 2016, at the Villum Research Station in Northeast Greenland. We used amplicon sequencing and qPCR targeting the 16S rRNA genes to assess the quantities and composition of the DNA and cDNA-level bacterial community. We found a clear seasonal variation in the atmospheric bacterial community, which is likely due to variable sources and meteorology. In early spring, the atmospheric bacterial community was dominated by taxa originating from temperate and Subarctic regions and arriving at the sampling site through long-range transport. We observed an efficient washout of the aerosolized bacterial cells during a snowstorm, which was followed by very low concentrations of bacteria in the atmosphere during the consecutive 4 weeks. We suggest that this is because in late spring, the long-range transport ceased, and the local sources which comprised only of ice and snow surfaces were weak resulting in low bacterial concentrations. This was supported by observed changes in the chemical composition of aerosols. In summer, the air bacterial community was confined to local sources such as soil, plant material and melting sea-ice. Aerosolized and deposited Cyanobacteria in spring had a high activity potential, implying their activity in the atmosphere or in surface snow. Overall, we show how the composition of bacterial aerosols in the high Arctic varies on a seasonal scale, identify their potential sources, demonstrate how their community sizes varies in time, investigate their diversity and determine their activity potential during and post Arctic haze.

## Introduction

Over the past 30 years the Arctic has warmed at approximately three times the rate as the entire globe, due to a series of positive feedbacks associated with, e.g., decreasing annual sea-ice cover thus effectively lowering the albedo, known as Arctic amplification (Lenton et al., 2008). This mechanism has been shown by measurements, observations as well as modeling (Holland and Bitz, 2003; Huang et al., 2019). An additional key factor involved in regulating the climate is the type and the level of cloud cover which affects the heat balance over a region. It is well established that aerosols can serve as cloud condensation nuclei (CCN) or ice nucleating particles (INPs) upon which cloud droplets and ice crystals can form in mixed phase clouds directly affecting cloud thickness, lifetime, and albedo (Pruppacher and Klett, 1997). An increasing number of studies indicate that different types of bioaerosols, including, e.g., aerosolized microorganisms or microbial compounds and fragments associated with mineral particles, are involved in cloud formation by acting as INPs whereby they may impact the Arctic and global climate (Murray et al., 2012, 2021; Šantl-Temkiv et al., 2019a,b, 2022; Wex et al., 2019).

To fully appreciate the processes involved in cloud and ice formation mediated by bioaerosols we must first obtain data on the quantity, diversity, and activity of the aerosolized microorganisms. Recent studies have addressed these questions mostly in temperate regions (Bowers et al., 2009; Temkiv et al., 2012; DeLeon-Rodriguez et al., 2013; Klein et al., 2016; Amato et al., 2017; Archer et al., 2021). A few studies have investigated these questions in the low and the high Arctic (Harding et al., 2011; Cuthbertson et al., 2017; Šantl-Temkiv et al., 2018; Tignat-Perrier et al., 2019). Harding et al. (2011) found that Cyanobacteria dominated the microbial community in snow samples in the Canadian high Arctic, while Cuthbertson et al. (2017) reported that Proteobacteria, Actinobacteria, and Firmicutes were the most abundant phyla in air collected above Svalbard and that 58 genera were consistently present in the air. Additionally, Šantl-Temkiv et al. (2018) reported that the atmospheric bacterial community in the low Arctic contained *1000* cells × m^–3^ of air and that these cells maintained a high 16S rRNA to 16S rRNA gene ratio – a measure of the cellular activity potential. They also found that the activity potential varied among taxa: it was low for Proteobacteria and high for Cyanobacteria and Actinobacteria. The “activity potential” has previously been used as a measure of a cells ability to rapidly respond to favorable environmental conditions by becoming metabolically active (Klein et al., 2016; Amato et al., 2017; Šantl-Temkiv et al., 2018; Bowsher et al., 2019). Tignat-Perrier et al. (2019) reported that only approx. 700 bacterial cells x m^−3^ of air were found in the atmosphere in the high Arctic and that this specific site had significantly lower atmospheric bacterial richness compared to mountain peaks, high altitude plateaus and marine remote sites. A study by Šantl-Temkiv et al. (2019a) addressed the abundance of biogenic INPs in spring and summer in the high Arctic and found that INPs that are active at high sub-zero temperatures (active at ≥−15°C) were present in both seasons but were more abundant in summer. They also found a correlation between the number of bacteria and biogenic INPs for aerosols collected in summer.

Currently, our understanding of Arctic microbial communities relies on short sampling series (Harding et al., 2011; Cuthbertson et al., 2017; Šantl-Temkiv et al., 2018). Thus, only a very coarse and limited picture of the abundance and temporality of bacterial aerosols in the Arctic can be drawn. Seasonality has a strong impact on the sources and types of aerosol particles in the high Arctic, due to distinct season-dependent transport mechanisms that feed aerosols into the Arctic atmosphere: (1) During the Arctic haze period which typically lasts from winter (December/January) to late spring (April/May), the dominant sources are situated at mid-latitudes (30–60°) and aerosols are transported over long distances also from mid-latitudes to the high Arctic (Shaw, 1995). This type of transport is associated with aerosols such as non-sea-salt-sulfate (nss-sulfate), organic compounds, soot, and acids of anthropogenic origin (Quinn et al., 2017; Dall Osto et al., 2018; Lange et al., 2019; Nielsen et al., 2019). (2) Outside the Arctic haze period, from April/May to November/December, the polar dome contracts due to the thermal stratification of the lower atmosphere at high latitudes and long-range transport of aerosol particles to the high Arctic becomes limited. Therefore, the contribution of aerosols from local regions dominates (Stohl, 2006; Bozem et al., 2019). Previous studies of this phenomenon did not address bacterial aerosols and it thus remains unclear how changes in large scale transport mechanisms affect bacterial communities in the high Arctic (Stohl, 2006; Bozem et al., 2019; Lange et al., 2019). Our sampling campaigns spanned the end of the Arctic haze period in early spring and the post Arctic haze period in late spring and summer. We combined qPCR data with high throughput sequencing of 16S rRNA gene transcripts and genes, meteorological and ceilometer data as well as the chemical analysis of aerosol composition to investigate whether the airborne bacterial communities vary with season, to determine their sources, community composition, diversity, and activity potential.

## Results and Discussion

### Richness and Composition of Airborne Bacterial Communities Reveal a Pristine Environment

**FIGURE 1:**
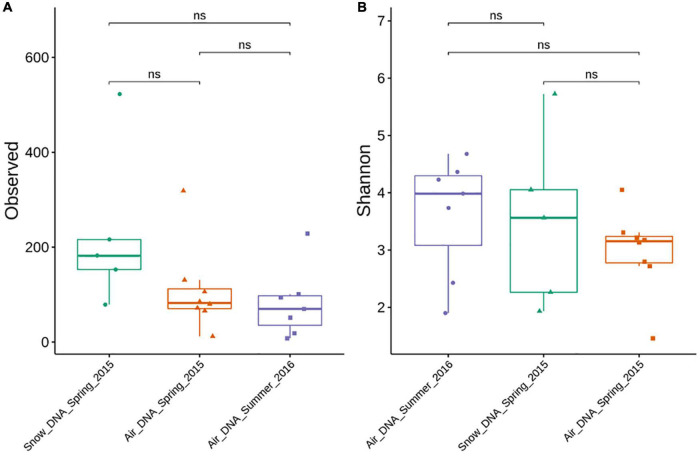
Alpha diversity (ASV richness and Shannon index) of DNA-level air and snow bacterial communities sampled during spring and summer. Significant differences between sample types were evaluated by a Kruskal-Wallis rank-sum test. Significance is denoted as: ns, nonsignificant as *p* > 0.05. **(A)** Observed ASV richness. **(B)** Shannon index.

The DNA and cDNA-level diversity of the bacterial communities were assessed in atmospheric and snow samples using rarefaction analysis. The analysis showed that the sequencing depth was sufficient to obtain a satisfactory coverage of diversity within individual samples as indicated by the fact that all rarefaction curves reach an asymptote (Supplementary Figure 1). In total, we detected 2988 unique amplicon sequence variants (ASVs) ranging from 8 to 523 ASVs in the individual samples (Figure 1A). The number of ASVs in freshly deposited surface snow samples was low compared to an earlier study of a depth profile from the same study area, which found that snow bacterial communities were composed of >4000 ASVs (Møller et al., 2013). The large number of ASVs reported in Møller et al. (2013) may reflect that they studied compacted, older snow, which may represent several deposition events. Their samples may also have been affected by postdepositional processes, such as selection and cell growth. Contrary, the number of ASVs in the air samples collected in spring and summer in this study closely resembled what had previously been reported in the Arctic (Cuthbertson et al., 2017; Šantl-Temkiv et al., 2018; Archer et al., 2021). Altogether, our results reflect the pristine nature of the Arctic atmospheric environment, characterized by the dilution and decay during long-range transport, few local sources, as well as scarce human presence and activity which together result in low bacterial diversity and abundance.

Although slightly more ASVs were observed in the snow samples compared to the air samples no significant difference was found based on a Kruskal–Wallis rank-sum test (Figure 1A). While most samples were characterized by a high evenness (Figure 1B), the Shannon diversity metrics was highly variable between samples ranging from <2 to >5. No statistically significant difference was found between the different sample types (Figure 1B).

**FIGURE 2:**
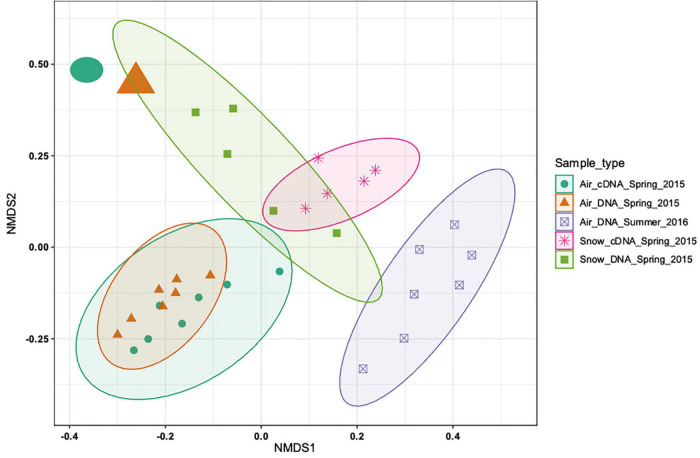
Non-metric multidimensional scaling (NMDS) based on the Bray-Curtis distance measure of 33 samples from 5 sample types of 2988 ASVs. No initial data transformation has been applied. The DNA-level air and snow bacterial communities sampled in spring and summer are shown and how close they cluster together. While most of the samples from the different sample types cluster closely together two samples (top left corner, sampled on the day of the snowstorm) cluster more closely with the snow samples than the other air samples.

The DNA and cDNA-level atmospheric communities in spring were similar both based on the non-metric multidimensional scaling analysis (NMDS; Figure 2) and on the analysis of similarities (ANOSIM, *R* = 0.06, *p* > 0.05). The DNA and the cDNA-level snow communities exhibited a slight overlap based on the NMDS analysis (Figure 2), but were significantly different (*R* = 0.632, *p* < 0.01). A clear separation between the DNA-level spring and summer air communities was found (Figure 2) that was supported by the ANOSIM analysis (*R* = 0.74, *p* < 0.001). Seasonal variability in the community composition of the near surface air community has previously been reported from Alpine environments in temperate regions (Bowers et al., 2013; Els et al., 2019). The differences between spring and summer atmospheric communities could be explained by the contraction of the polar dome at the end of spring (Bozem et al., 2019). This shift leads to decreased long-range aerosol transport and to an increased importance of local sources once snow and ice melt, exposing terrestrial and marine surfaces (Stohl, 2006; Dall Osto et al., 2018). On average, there was a clear separation between the bacterial community composition in snow and air (*R* = 0.59, *p* < 0.001) collected in spring. However, the spring air community sampled just before the snowstorm (enlarged circle and triangle, top left corner in Figure 2) was more similar to the snow bacterial community than to the remaining air bacterial communities. Overall, our analysis shows that there is a clear seasonal separation between the air bacterial communities as well as a separation between the air and snow bacterial communities.

### Seasonal Taxonomic Diversity Is Linked to Available Sources

**FIGURE 3:**
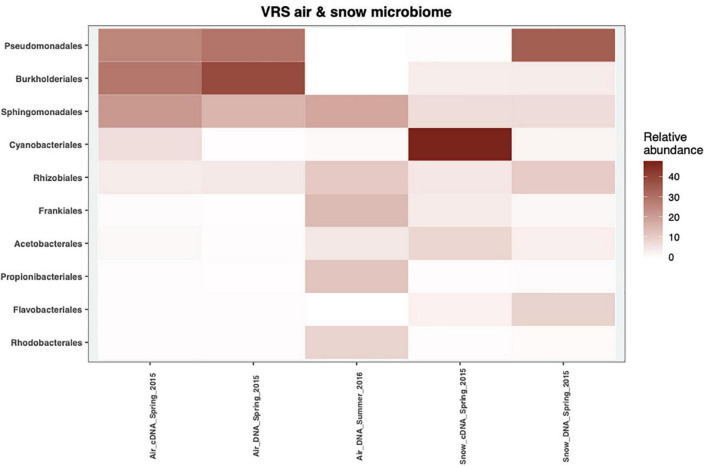
The mean relative abundance of the top 10 most abundant orders in 2015 and 2016 of the cDNA-level and DNA-level bacterial communities from snow and air.

Five phyla dominated the DNA and cDNA-level snow and air bacterial communities both in spring and summer (Supplementary Figure 2). The Proteobacteria phylum was the most abundant phylum in all samples, accounting for at least 32% of the cDNA-level snow community and up to 95% of the DNA-level atmospheric community in spring (Supplementary Figure 2). The second most abundant phylum was Cyanobacteria, ranging from <1% of the DNA-based atmospheric community in spring up to 50% of the cDNA-level snow community (Supplementary Figure 2). Actinobacteriota accounted for <1% in the DNA- level spring atmospheric community and up to 42% in the summer atmospheric community (Supplementary Figure 2). The Bacteroidota accounted for 15% in the DNA-level snow community and 6% in the cDNA-level snow community, respectively, but <2% in all the other samples. Finally, Firmicutes accounted for 5% in the DNA-level summer atmospheric community but <2.5% in other samples. These results are comparable to other studies of air and precipitation both in the Arctic- (Møller et al., 2013; Cuthbertson et al., 2017; Šantl-Temkiv et al., 2018) and in the temperate region (Bowers et al., 2009; Šantl-Temkiv et al., 2013; Amato et al., 2017). Members of Pseudomonadales dominated both the DNA-level snow community as well as the spring atmospheric community at the order level (25–35%), but were missing in the summer atmospheric community as well as in the cDNA-level snow community (Figure 3). Amato et al. (2015) proposed that members of this order get aerosolized and thereafter trigger their own deposition from the atmosphere by producing ice-nucleation proteins (INpro) thereby inducing ice formation in clouds. All spring atmospheric communities had a high abundance of Burkholderiales and Sphingomonadales, accounting for 29–39% and 18–22% of the communities, respectively. Further, the abundance of Sphingomonadales was also high (19%) in the summer atmospheric communities. The Burkholderiales are widespread in the Arctic and are considered a typical member of the Arctic soil community (Malard and Pearce, 2018) while the Sphingomonadales have been associated with both the Arctic air (Cuthbertson et al., 2017) and the snow bacterial community in the Arctic (Møller et al., 2013). The cDNA-level snow community was dominated by the order Cyanobacteriales (47%). The Cyanobacteriales are well known to occupy both freshwater and saltwater niches as well as snow, ice and cryoconite holes in the Arctic (Møller et al., 2013; Boetius et al., 2015; Šantl-Temkiv et al., 2018). Frankiales, Acetobacterales, Propionibacteriales, and Rhodobacterales were present at low abundance in the spring atmospheric communities (>1.5%), while present at larger relative abundance in the summer atmospheric communities (14, 4.5, 12, and 9%), respectively. Interestingly, Frankiales that have previously been found in the Arctic can fix nitrogen and often live in symbiosis with actinorhizal plants (Benson and Silvester, 1993; Nash et al., 2018). Klarenberg et al. (2020) showed that members of the Acetobacterales dominate the bacterial community of the thalli of lichens that prevail on 8% of the world’s land surface, mainly in Arctic and Antarctic regions (Beckett et al., 2013). Finally, both Propionibacteriales and Rhodobacterales were found in the active layer of thawing permafrost soil in the Arctic (Inglese et al., 2018). Overall, we suggest that the spring atmospheric bacterial community assembles from both distant as well as local sources such as snow, sea ice, and sea ice leads. The shift in bacterial community composition between spring and summer is likely explained by a switch from distant to local sources such as sea water, permafrost soil, plants, or lichens.

### Taxa Within the Bacterial Communities With an Elevated Activity Potential

**FIGURE 4:**
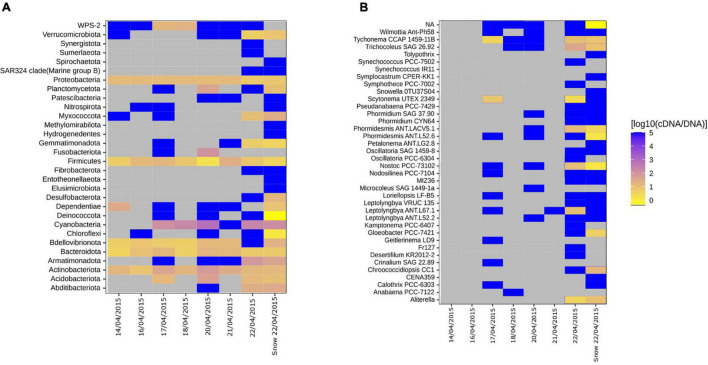
Heatmaps showing the ratio between the proportion of a clade in the cDNA-level and in DNA-level community for individual air samples and mean snow samples. **(A)** All phyla. **(B)** Genera associated with the Cyanobacterial phyla. Gray tiles indicate that the specific taxon is not present in neither cDNA nor DNA-level community. Bright yellow indicate that the taxa is present in the DNA-level community but not in the cDNA-level community. Dark blue tiles indicate that the taxon is present in the cDNA-level community but not in the DNA-level community, which have previously been referred to as “phantom taxa” (Klein et al., 2016).

We assessed the activity potential of the air and snow bacterial communities based on cDNA:DNA (16S rRNA:16S rRNA gene) ratio. We found that Cyanobacteria, Actinobacteriota, Firmicutes, Proteobacteria, and Bacteroidota showed a high 16S rRNA:16S rRNA gene ratio in nearly all samples (Figure 4A). Specific taxa like Clostridiales, Rubrobacteridae, and Cyanobacteria with an elevated activity potential have been reported for the airborne bacterial communities in the low Arctic (Šantl-Temkiv et al., 2018). In our study, cells belonging to members of Cyanobacteria phylum showed the highest activity potential (Figure 4). This agrees with what other studies have shown. Both in temperate regions (Dillon et al., 2020) as well as the Arctic (Šantl-Temkiv et al., 2018) Cyanobacteria maintain a high ribosomal content while aerosolized. The authors suggest that the high content of ribosomes in aerosolized cells allow for a faster response to atmospheric stress, e.g., UV, desiccation and freezing (Dillon et al., 2020) as well as to the conditions after their deposition on ground (Šantl-Temkiv et al., 2018). Of the 38 cyanobacterial genera (Figure 4B), the genera *Tychonema, Phormidesmis, Nostoc, Aliterella*, and *Trichocoleus* had a high activity potential both in air and snow (Figure 4B). *Tychonema* sp. had a 16S rRNA:16S rRNA gene ratio between 8.13 and 10 in samples where it was present. *Tychonema* are filamentous freshwater cyanobacteria that were previously isolated from microbial freshwater mats in the high Arctic (de los Ríos et al., 2015). *Phormidesmis* sp. and *Nostoc* sp., which had 16S rRNA:16S rRNA gene ratios between 3.02 and 12.88 and 10.72 and 26.3, respectively, are common in the cryosphere, e.g., on the Greenlandic ice sheet, on glaciers and in fresh water melt ponds (Liengen and Olsen, 1997; Chrismas et al., 2016). *Aliterella* had an activity potential between 3.47 and 144.54 and has previously been found in coastal Antarctica (Jung et al., 2020), while *Trichocoleus SAG 26.92* had an activity potential ranging from 8.51 to 38.02 and is usually associated with desert soil crust (Muhlsteinova et al., 2014). Interestingly, *Tychonema CCAP 1459-11B* was among the top 10 most abundant ASVs in both snow and air on the day of the snowstorm (Supplementary Table 2) and maintained a high activity potential in both air and snow, which implies that this genus may be an important tracer for the local aerosol source that triggered the storm.

### Airborne Bacterial Community Wash-Out During a Snowstorm

**FIGURE 5:**
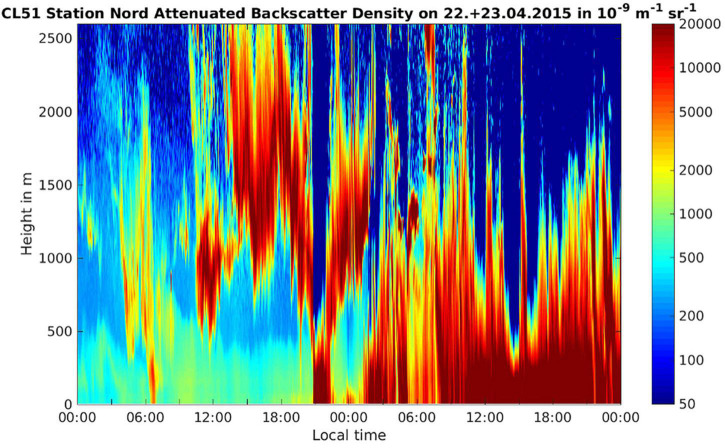
Attenuated backscatter density (units 10^− 9^ sr^− 1^ m^− 1^) on the day of the onset of the storm (22 April 2015) and the following day from the ground up to 2,500 m height. The clouds associated with the storm are shown by red/brown colors indicating high values of the attenuated backscatter. It can be seen that the cloud systems move in at around 12 pm on 22 April, reaches ground level at around 21 pm, then ascends up >500 m for approx. 3 h before descending and then remains in contact with the ground for the whole of the 23rd of April.

A snowstorm event occurred during our sampling campaign in spring (April 22nd, Figure 5). At the onset of the storm the wind direction changed from Southwest to Northwest and the wind speed increased from <5 to 15.7 m⋅s^–1^ (Supplementary Figure 3). Atmospheric bacterial community that was rather stable over a week prior to the snow storm, changed dramatically on the day of the storm (Figure 2). Increased wind speed can affect bioaerosol composition and number through two major mechanisms. On the one hand side, the dry deposition rate of particles is enhanced with increasing wind speed. An increase in wind speed from 2 to 20 m⋅s^–1^ was modeled to cause an 100-fold increase in deposition velocity for 1 μm sized particles, which is a typical size for bacterial cells. On the other hand, high wind speed has been shown on several occasions to enhance aerosolization through increased mechanical force acting on the surfaces, including water, snow and soil (Jones and Harrison, 2004; Qi et al., 2005). We therefore suggest that the rather stable bacterial community, that we observed prior to the snow storm, was deposited by enhanced dry deposition due to high wind speeds just before the snowstorm, while new cells were aerosolized simultaneously from local sources.

The temperature at ground level during the snow storm was between −13 and −21°C. At conditions of low surface net radiation in the Arctic winter and spring, temperature inversions have been observed frequently with temperatures in the troposphere rising with height (Serreze et al., 1992). Due to the thick snow cover at the Villum Research Station during spring the surface net radiation was low and therefore a temperature inversion was plausible, implying that cloud top temperatures would likely not support homogeneous freezing but instead presence of INPs was necessary to induce freezing and precipitation. This is supported by observations showing that in April >65% of the clouds in the Arctic were mixed phase clouds (Intrieri, 2002). By analyzing the attenuated backscatter observed with a ceilometer at the Villum Research Station, we could estimate the cloud base height on the day of the snowstorm (Figure 5). The attenuated backscatter shows a cloud that formed at around noon with a cloud base ∼500 m above ground level (Figure 5). Based on the attenuated backscatter, the cloud base may have descended to ground level at around 9 pm and therefore the aerosols in the boundary layer closest to the ground level, including bacterial cells, entered the cloud, which coincided with the onset of the snowstorm (Figure 5). Alternatively, the attenuated backscatter at the ground level comes from the snow fall. We propose two alternative scenarios that are consistent with the data: (1) Particles from the cryosphere, e.g., snow crystals, were aerosolized by high wind speeds and blown into the cloud together with the snow bacterial community. Snow crystals were previously reported to act as ice nuclei, triggering glaciation and ultimately precipitation (Lloyd et al., 2015; Beck et al., 2018). (2) Specific members of the air bacterial community triggered the snowfall due to biological ice nucleation. INP were previously linked to biogenic sources at the Villum Research Station (Šantl-Temkiv et al., 2019a). Surprisingly, we did not find any 16S rRNA gene sequences that are similar to those found in ice-nucleation active *Pseudomonas, Xanthomonas, Pantoea*, or *Erwinia* species (Failor et al., 2017) within the atmospheric communities just before the snowstorm or in snow samples. These findings are supported by a study of ice-nucleation activity (INA) in bacteria and yeast isolates from aerosol and precipitation samples collected during the same period (Šantl-Temkiv et al., 2019a). In this study, no INA microorganisms were found using a culture-dependent approach. Alternatively, other than the well known INA microorganisms are present, e.g., *Lysinibacillus parviboronicapiens*, microalgae or Cyanobacteria (e.g., *Microcysti*s sp.) (Tesson and Santl-Temkiv, 2018; Failor et al., 2022).

**FIGURE 6:**
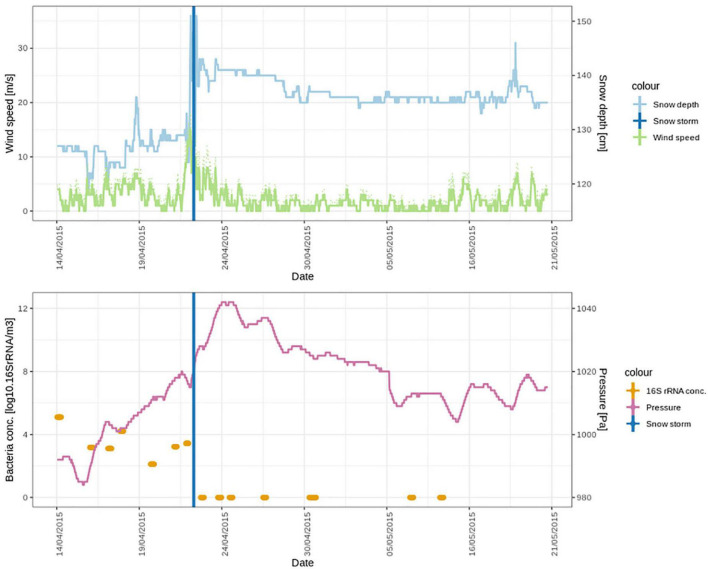
Meteorological conditions and bacterial cell concentrations in April and May 2015, during the period of the snowstorm (22nd of April). In the lower panel, the bacterial cell concentrations are detectable over a week (seven air samples) until the snowstorm. After the snowstorm, no bacterial cells could be detected in the air.

The storm was accompanied by snowfall (Figure 6 top panel). We investigated the similarities between the DNA and the cDNA-level atmospheric bacterial community on the day of the snowstorm and in the freshly fallen snow. Out of a total 1129 ASVs in the DNA-level community, 50 ASVs were shared. These ASVs accounted for 19.1% of the DNA-level community (Supplementary Figure 4A). However, when looking at the cDNA-level community 88 ASVs out 1114 ASVs where shared and these accounted for more than 40% of the DNA and cDNA-level community abundance. Taxa which stem mainly from the Sphingomonadales order were found in the 10 top most abundant ASVs from the DNA-level community which were shared between the atmosphere and the snow (Supplementary Table 1). While more ASVs from the Cyanobacteriales were shared between the atmosphere and snow in the cDNA-level community (Supplementary Table 2). However, 24 different families were found both in the DNA and the cDNA-level community which were shared between the atmosphere and snow on the day of the snowstorm. This observation indicates that the bacterial community in the atmosphere was washed out during the snowstorm through wet deposition, which may be due to in-cloud processes, including bacterial cells acting as CCN or being scavenged by cloud droplets and ice particles. Scavenging of submicron to micron sized microbial aerosols has also been experimentally demonstrated below clouds using simulated rainfalls (Hanlon et al., 2017; Moore et al., 2020). A complete washout of the atmospheric community is additionally supported by the fact that while atmospheric bacterial cell concentrations varied between 1.3×10^2^ and 1.3×10^5^ cells × m^–3^ of air on the days before the storm, we could not detect any airborne bacterial cells after the snowstorm (Figure 6 lower panel).

**FIGURE 7:**
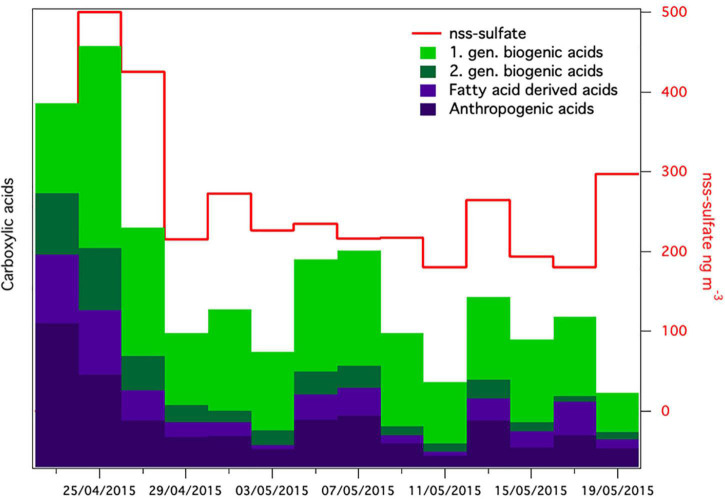
Relative concentrations of groups of carboxylic acids together with non-sea-salt sulfate [nss-sulfate in aerosol samples (nss-sulfate was not analyzed in the first sample)].

The meteorological conditions were significantly different after the snowstorm compared to before the snowstorm. Wind speed after the storm was significantly lower (2.2 ± 1.8 m⋅s^–1^) than before the storm (4.22 ± 4.2 m⋅s^–1^) (*t* = 8.9577 df = 881, *p* < 0.0001). Pressure (*t* = 11.2891, df = 881, *p* < 0.0001) was significantly higher. Supplementary Figure 3 shows wind roses during spring that depict the wind direction before, during and after the storm, which were all significantly different (*t* = 3.4380, df = 881, *p* < 0.001). Before the storm, strong winds from southwest (SW) dominated at the Villum Research Station. After the storm the wind direction still was predominantly from SW but strong winds came from the North (Supplementary Figure 3C). Concentrations of non-sea-salt sulfate (nss-sulfate), as well as anthropogenic acids, which are common tracers of long-range transport (Nguyen et al., 2013; Hansen et al., 2014; Massling et al., 2015), were high before and around the snowstorm and decreased during the 4 weeks after the snowstorm (Figure 7). This suggests that the Arctic haze period, which is associated with significant contributions of long-range transport from mid-latitudes to aerosol populations in the Arctic, had stopped around the time of the snowstorm. This is further supported by a study from the same period investigating the biogenic and anthropogenic sources of soot aerosols that also found that long-range transport had stopped in the late spring (Nielsen et al., 2019). We propose that after the wash-out, the atmospheric bacterial community could not re-establish and reach detectable concentrations due to ceased long-range transport in combination with extensive ice and snow cover, which reduced aerosolization from local sources.

### Conclusion and Future Perspectives

Our study shows that the atmospheric and snow bacterial communities in the high Arctic consist of a low number of ASVs compared to the communities found in the atmosphere over temperate regions. We observed a clear seasonal variation in the atmospheric bacterial community. We suggest that the early spring airborne bacterial community is dominated by cells arriving via long-range transport from temperate and sub-Arctic region with a contribution from local sources, i.e., the cryosphere. The late spring and the summer air communities are dominated by cells that stem from local and regional sources such as soil, plant material, open sea or marginal sea-ice. We found that Cyanobacteria showed a high activity potential while aerosolized as well as after being deposited in the snow during spring. We report the first observation of a complete washout of aerosolized bacteria into the snow, which was likely a combination of in-cloud processes, including bacterial cells acting as CCN or being scavenged by cloud droplets and ice particles, and bellow-cloud scavenging of bacterial cells by snowfall. Finally, we show that the concentration of the airborne bacteria was below the detection limit of our sampling and analytical methods for at least 4 consecutive weeks after the snowstorm. By using chemical analysis, we demonstrate that soon after the snow storm the Arctic haze period and therefore the predominant contribution of the long-range dissemination ended, leaving the cryosphere as the only available source for a new airborne bacterial community to re-establish. To reveal whether our observations can be generalized, the frequency and length of field campaigns as well as the number of sampling stations should be increased in future studies. In addition, to obtain a more in depth understanding of how the Arctic haze period and wash-out events affect the assembly as well as the atmospheric role of airborne bacteria communities, future measurements should complement quantitative and qualitative data on microbial communities with studies of INPs, chemical aerosol measurements and cloud dynamics.

## Materials and Methods

### Sampling Location, Design and Collection

Sampling location, design, and collection has been previously described in detail (Šantl-Temkiv et al., 2019a). In short, air samples were collected in spring 2015 and summer 2016 in proximity of the Villum Research Station (VRS, Station Nord, 81°36′ N, 16°40′ W) (Supplementary Tables 3, 4). We collected 25 aerosol samples, using a high-flow-rate impinger Kärcher DS5800 powered by a generator (Šantl-Temkiv et al., 2017) and low-flow-rate filter sampling on cellulose nitrate membrane filters (0.2 μm pore size, Whatman, Germany). We collected a negative control prior to each sample collected with the high-flow-rate impinger as well as two control samples for the low-flow-rate filter sample as previously described (Šantl-Temkiv et al., 2019a).

Additionally, fifteen surface snow samples (top 5–10 cm of snow) were collected in a 60-km transect in April 2015 into sterile polystyrene bags. Only surface snow was collected in order to sample particles deposited with a fresh precipitation event. These snow samples are representative of a single precipitation event. The snowfall occurred a few days prior to sampling the snow (April 22nd). The snow was transported back to the laboratory and melted at room temperature. The snowmelt was concentrated on a Sterivex filter and RNA later was added to prevent any changes in activity during storage (0.2 μm pore size, Sigma-Aldrich, Germany). Air samples used for 16S rRNA transcript analysis were collected in a high-salt solution (Lever et al., 2015; Šantl-Temkiv et al., 2018) and stored in the presence of RNA later to prevent any changes in activity. All samples were stored at −20°C prior to the analysis. Supplementary Table 3 gives an overview of all samples, including the sampling time, rate, and volume.

### DNA/RNA Extraction and Quantitative Polymerase Chain Reaction

DNA was extracted from aerosol samples collected on filters in summer (Xiao et al., 2017; Šantl-Temkiv et al., 2019a) while DNA/RNA were co-extracted from the aerosol and snow samples collected during the Arctic haze period in spring as described earlier (Lever et al., 2015; Šantl-Temkiv et al., 2019a). Quantitative Polymerase Chain Reaction (qPCR) using an MX3005p qPCR instrument (Agilent, Santa Clara, CA, United States) was performed to quantify the amount of bacterial 16S rRNA gene copies (DNA) and transcripts (cDNA) in 38 DNA and cDNA extracts. We targeted partial 16S rRNA gene sequence using universal primers Bac908F (5′-AAC TCA AAK GAA TTG ACG GG-3′) and Bac1075R (5′-CAC GAG CTG ACG ACA RCC-3′) (Ohkuma and Kudo, 1998) as described earlier (Lever et al., 2015). All sampling controls were treated in the same way as the samples. Only samples which were higher in their 16S rRNA copy number compared to the corresponding control were selected for amplicon sequencing. This resulted in 15 air samples and five snow samples sequenced at the DNA level as well as eight air samples and five snow samples sequenced at the cDNA level (Supplementary Tables 3, 4).

### Amplicon Sequencing

The hypervariable region V3 and V4 of the 16S rRNA gene was amplified from DNA and cDNA samples with primers Bac341F (5′-CCT ACG GGN GGC WGC AG-3′) and Bac805R (5′-GAC TAC HVG GGT ATC TAA TCC-3′). The 16S rRNA gene amplification was performed according to a modified Illumina protocol (16S Metagenomic Sequencing Library Preparation, Part # 15044223 Rev. B). The PCR mixture contained 2–5 μl template DNA, 2 × KAPA HiFi HotStart polymerase (Kapa Biosystems, Inc., Wilmington, MA, United States), 0.2 μM forward primer, 0.2 μM reverse primer, and BSA (4 g/L). Two microliters of template DNA was used when amplifying samples obtained by the high-flow-rate impinger and snow samples and 5 μL of template DNA was used for samples collected on filters. The thermal cycling was run with an initial denaturation step at 95°C for 3 min, 30 cycles with denaturation at 95°C for 30 s, annealing at 55°C for 30 s, elongation at 72°C for 30 s and a final elongation at 72°C for 5 min. The PCR products were cleaned using 30 μl AMPure XP magnetic beads. The second PCR incorporated the Illumina overhang adaptors in absence of BSA. The PCR was run for 10 cycles using the same conditions as for the first PCR. The product was cleaned with 20 μl AMPure XP beads. The third PCR was run to incorporate the Nextera XT Index primers. Each reaction contained 2.5 μl of each index primers (N7XX and S5XX), 12.5 μl KAPA HiFi HotStart ReadyMix and 5 μl dH2O. The PCR reaction was run for 8 cycles using the same conditions as for the first two PCR and the PCR product was cleaned with 56 μl AMPure XP beads. The PCR products were quantified using a Quant-iT™ dsDNA BR assay kit on a FLUOstar Omega fluorometric microplate reader (BMG LABTECH, Ortenberg, Germany), diluted and pooled together in equimolar ratios. The pool was quantified using the Quant-iT™ dsDNA BR assay kit on a Qubit fluorometer (Thermo Fisher Scientific, Waltham, MA, United States) and then sequenced on the Illumina MiSeq platform (Illumina, San Diego, CA, United States) which produces two 300-bp long paired-end reads.

### Bioinformatic Analysis

Bioinformatic analyses were performed in RStudio 4.1.0. 16S rRNA amplicons from spring 2015 and summer 2016 were initially analyzed individually following the DADA2 workflow for Big Data^1^. Primer and adapter sequences were trimmed from the raw reads using cutadapt 0.0.1 (Martin, 2011). Forward and reverse read quality were plotted with the plotQualityProfile function from DADA2 1.21.0 (Callahan et al., 2016). Based on the read quality a trimming of 240 and 190 bp were set for the forward and reverse reads, respectively, using FilterAndTrim, according to their quality (Callahan et al., 2016). The fastq files were randomly subsampled to the lowest read number using the ShortRead package 1.48.0 (Morgan et al., 2009), resulting in 11,193 reads per sample. The subsampling allows for a more accurate comparison of the richness of the different samples. Error models were built for the forward and reverse reads, followed by dereplication and clustering into ASVs (Callahan et al., 2017) with DADA2. The denoised forward and reverse reads were merged using the function mergePairs with default parameters with a minimum overlap of 12 nucleotides, allowing 0 mismatches. Sequence tables were made with the function makeSequenceTable and the two sequence tables from 2015 to 2016 were combined using the mergeSequenceTables function, as proposed in the DADA2 workflow for Big Data (see text footnote 1). ASVs shorter than 402 and longer than 431 nucleotides were removed from the dataset and chimeras were removed using the removeBimeraDenovo function. Taxonomic assignment was accomplished using the naive Bayesian classifier against the SILVA ribosomal RNA gene database v138 (Quast et al., 2012) with the assignTaxonomy function from DADA2, and species assignment was performed with the assignSpecies function from DADA2. ASVs mapped to mitochondria and chloroplasts were removed from the dataset. We used observed richness (number of ASVs) in the samples vs. negative controls as an additional criterium for excluding samples with too low biomass: (i) in case of samples collected with the high-flow-rate impinger, which have a somewhat higher level of contamination, only samples containing ≥50 ASVs were used for further analysis (14 out of 15) and (ii) in case of samples collected on filters, samples containing ≥10 ASVs were used (7 out of 7). Samples were decontaminated using the prevalence method (Threshold = 0.1) from the Decontam package (Davis et al., 2018). Statistical tests and visualization of the data was performed with phyloseq (McMurdie and Holmes, 2013) and microeco (Liu et al., 2021).

### Chemical Composition of Aerosols

Aerosol samples (48 h) were collected on quartz fiber filters using a high-volume sampler (DHA-80, Digitel, Germany). Carboxylic acids were extracted and analyzed by ultrahigh performance liquid chromatography coupled to quadrupole time-flight-mass spectrometry (UHPLC-QTOF-MS) using a previously published method (Hansen et al., 2014). The following groups of carboxylic acids were quantified: anthropogenic acids (adipic acid, phthalic acid, and pimelic acid), first-generation biogenic acids derived from monoterpenes (terpenylic acid, pinic acid, and pinonic acid), second-generation biogenic acid (3-methyl butane tricarboxylic acid), and fatty-acid derived acids (suberic acid and azelaic acid). Due to a problem with the inlet of the high-volume sampler, only a fraction of fine aerosols was collected, and the concentrations are thus presented as relative values. Inorganic ions were quantified by ion chromatography for determination of non-sea-salt (nss) sulfate.

### Ceilometer Data

Profiles of attenuated backscatter were obtained from observations by a vertically pointing Vaisala CL51 laser diode ceilometer. It is a ground based remote sensing instrument that emits pulses of light with a wavelength of 905 nm at a frequency of 10 MHz. During the passage through the atmosphere, the light pulses are scattered by aerosols particles, cloud/rain droplets. The backscattered signal is proportional to the concentration of the particles, therefore the instrument is often use for the detection of clouds and fog, both are having high values of the backscatter. As the light pulses travel through the air, small fractions of the backscatter light reach the ceilometer and are recorded. The travel times of the pulses are transformed to height and in this way, after compensating for the height square decrease of the backscatter, an attenuated profile of the backscattered signal is obtained. The profile range resolution for the instrument at Station Nord/Villum Research Station is set to 10 m and with a maximum range of 7,700 m.

## Data Availability Statement

The data presented in this study are deposited in the European Nucleotide Archive under the accession number PRJNA844291.

## Author Contributions

TŠ-T, KF, and AM designed the research project. TŠ-T, MG, and AM supervised the sample collection. TŠ-T collected the samples during the Arctic Haze period and supervised the microbial analysis. MG supervised the chemical analysis. S-EG supervised the ceilometer measurements and analyzed the ceilometer data. LJ performed the bioinformatic analysis and drafted the manuscript. LJ, KF, and TŠ-T wrote the manuscript with contributions from other co-authors. All authors contributed to the article and approved the submitted version.

## Conflict of Interest

The authors declare that the research was conducted in the absence of any commercial or financial relationships that could be construed as a potential conflict of interest.

## Publisher’s Note

All claims expressed in this article are solely those of the authors and do not necessarily represent those of their affiliated organizations, or those of the publisher, the editors and the reviewers. Any product that may be evaluated in this article, or claim that may be made by its manufacturer, is not guaranteed or endorsed by the publisher.

## Footnotes

1
https://benjjneb.github.io/dada2/bigdata.html
