# Supplementary material for: Seasonal Variation of the Atmospheric Bacterial Community in the Greenlandic High Arctic Is Influenced by Weather Events and Local and Distant Sources
Source: Front Microbiol. 2022 Jul 8;13:909980. doi: 10.3389/fmicb.2022.909980 (PMC9307761; doi:10.3389/fmicb.2022.909980)
Supplement: Supplementary file 1 [file Data_Sheet_1.docx]

Seasonal Variation of the Atmospheric Bacterial Community in the Greenlandic High Arctic Is Influenced by Weather Events and Local and Distant Sources

Seasonal Variation of the Atmospheric Bacterial Community in the Greenlandic High Arctic Is Influenced by Weather Events and Local and Distant Sources

Lasse Z. Jensen^1,2,3^, Marianne Glasius^4^, Sven-Erik Gryning^5^, Andreas Massling^3,6^, Kai Finster^1,7^, Tina Šantl-Temkiv^1,2,3,7^

^1^Department of Biology, Microbiology Section, Aarhus University, Ny Munkegade 116, 8000 Aarhus, Denmark

^2.^ Arctic Reseach Center, Aarhus University, Ny Munkegade 116, 8000 Aarhus, Denmark

^3^ iCLIMATE Aarhus University Interdisciplinary Centre for Climate Change, Frederiksborgvej 399, Roskilde, Denmark

^4^Department of Chemistry, Aarhus University, 8000 Aarhus C, Denmark

^5^DTU Wind Energy, Technical University of Denmark, Risø Campus, Roskilde, Denmark

^6^Department of Environmental Science, Aarhus University, 4000 Roskilde, Denmark

^7^Stellar Astrophysics Centre, Department of Physics and Astronomy, Aarhus University, Ny Munkegade 120, 8000 Aarhus, Denmark

*** Correspondence:**Corresponding Author
Temkiv@bio.au.dk

Keywords: Bioaerosols, Atmospheric bacterial community_,_ Arctic haze, Microbial activity. Ice nucleation

Figure S1 Rarefaction curves of 33 samples grouped into 5 different sample types. To avoid inflation of the rarefaction analysis, the dataset was down sampled to 11193 reads per sample before analysis.

Figure S2 The mean relative abundance of the top 5 most abundant phyla in 2015 and 2016 of the cDNA and DNA-level bacterial communities from snow and air.


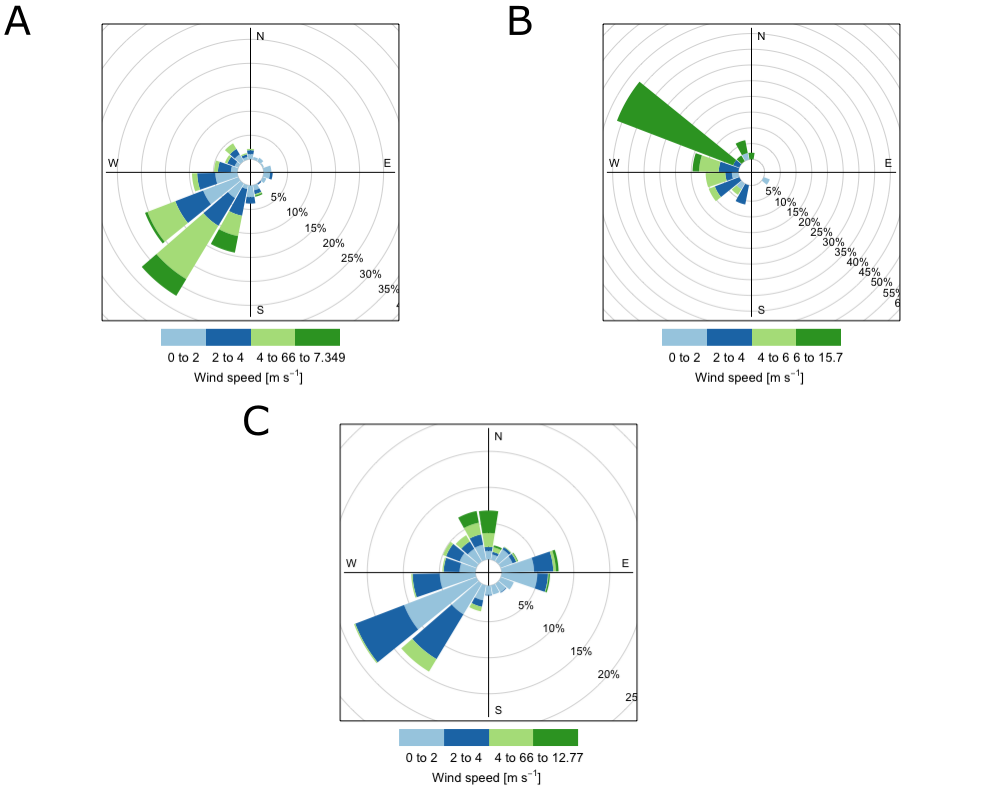


Figure S3 Wind roses showing the frequency of different wind directions at Villum Research Station in April and May (A) before the storm dominated by winds from southwest (SW), (B) Under the storm dominated by strong winds from northwest (NW) and (C) after the storm the wind direction changed back to SW although still with some strong winds from north.

Figure S4 Venn diagram showing the shared and unique ASVs of the A) DNA-level and B) cDNA-level air bacterial community just before the snowstorm and the snow bacterial community after the snowstorm. The integer number is the number of ASVs which are either unique or shared between the air and snow. Numbers in brackets show how much of relative abundance from the whole dataset that the unique or shared ASVs make up.

**Table S.1** Top 10 most abundant shared ASVs between the total (DNA) air and snow bacterial community on the day of the snowstorm (22^nd^ of April) and the contribution to relative abundance within the sample type. Coloured names denote the same ASV

| **Top 10 ASVs in the Air bacterial community** | **Relative ASV abundance (%)** | |
| --- | --- | --- |
| **Taxonomic annotation** | **Air** | **Snow** |
| Proteobacteria; Alphaproteobacteria; Rhizobiales; Rhizobiaceae; Allorhizobium-Neorhizobium-Pararhizobium-Rhizobium | 2.05 | 6.93 |
| Proteobacteria; Alphaproteobacteria; Sphingomonadales; Sphingomonadaceae; Sphingomonas | 1.37 | 0.87 |
| Proteobacteria; Alphaproteobacteria; Sphingomonadales; Sphingomonadaceae; Sphingomonas | 1.00 | 0.03 |
| Actinobacteriota; Actinobacteria; Frankiales | 0.81 | 0.49 |
| Proteobacteria; Alphaproteobacteria; Sphingomonadales; Sphingomonadaceae; Sphingomonas | 0.58 | 0.09 |
| Proteobacteria; Alphaproteobacteria; Acetobacterales; Acetobacteraceae; Acidiphilium | 0.55 | 0.27 |
| Cyanobacteria; Cyanobacteriia; Cyanobacteriales; Phormidiaceae; Tychonema CCAP 1459-11B | 0.51 | 0.47 |
| Proteobacteria; Alphaproteobacteria; Sphingomonadales; Sphingomonadaceae; Sphingomonas | 0.50 | 0.59 |
| Proteobacteria; Alphaproteobacteria; Sphingomonadales; Sphingomonadaceae; Sphingomonas | 0.47 | 0.28 |
| Proteobacteria; Alphaproteobacteria; Sphingomonadales; Sphingomonadaceae; Sphingomonas | 0.46 | 0.14 |
| **Top 10 ASVs in the Snow bacterial community** | **Relative ASV abundance (%)** | |
| **Taxonomic annotation** | **Snow** | **Air** |
| Proteobacteria; Alphaproteobacteria; Rhizobiales; Rhizobiaceae; Allorhizobium-Neorhizobium-Pararhizobium-Rhizobium | 6.93 | 2.05 |
| Proteobacteria; Alphaproteobacteria; Rhizobiales; Xanthobacteraceae; Afpia | 3.78 | 0.33 |
| Bacteroidota; Bacteroidia; Chitinophagales; Chitinophagaceae; Asinibacterium | 2.76 | 0.23 |
| Proteobacteria; Alphaproteobacteria; Sphingomonadales; Sphingomonadaceae; Novosphingobium | 1.56 | 0.19 |
| Proteobacteria; Gammaproteobacteria; Gammaproteobacteria Incertae Sedis; Unknown Family; Candidatus Ovatusbacter | 1.32 | 0.24 |
| Proteobacteria; Alphaproteobacteria; Sphingomonadales; Sphingomonadaceae; Novosphingobium | 1.11 | 0.17 |
| Proteobacteria; Alphaproteobacteria; Sphingomonadales; Sphingomonadaceae; Sphingomonas | 0.87 | 1.37 |
| Proteobacteria; Alphaproteobacteria; Caulobacterales; Caulobacteraceae | 0.84 | 0.13 |
| Proteobacteria; Alphaproteobacteria; Sphingomonadales; Sphingomonadaceae; Sphingomonas | 0.59 | 0.50 |
| Actinobacteriota; Actinobacteria; Frankiales | 0.49 | 0.81 |

**Table S.2** Top 10 most abundant shared ASVs between the active (cDNA) air and snow bacterial community on the day of the snowstorm (22^nd^ of April) and the contribution to relative abundance within the sample type. Coloured names denote the same ASV

| **Top 10 ASVs in the active air bacterial community** | **Relative ASV abundance (%)** | |
| --- | --- | --- |
| **Taxonomic annotation** | **Air** | **Snow** |
| Cyanobacteria; Cyanobacteriia; Cyanobacteriales; Phormidiaceae; Tychonema CCAP 1459-11B | 6.56 | 3.16 |
| Cyanobacteria; Cyanobacteriia; Cyanobacteriales; Chroococcidiopsaceae | 2.67 | 5.09 |
| Cyanobacteria; Cyanobacteriia; Cyanobacteriales; Nostocaceae; Scytonema UTEX 2349 | 2.30 | 0.59 |
| Proteobacteria; Alphaproteobacteria; Acetobacterales; Acetobacteraceae; Acidiphilium | 1.97 | 1.62 |
| Cyanobacteria; Cyanobacteriia; Cyanobacteriales; Phormidiaceae; Trichocoleus SAG 26.92 | 1.83 | 0.12 |
| Cyanobacteria; Cyanobacteriia; Phormidesmiales; Nodosilineaceae; Nodosilinea PCC-7104 | 1.74 | 0.70 |
| Cyanobacteria; Cyanobacteriia; Cyanobacteriales; Chroococcidiopsaceae; Aliterella | 1.45 | 1.55 |
| Proteobacteria; Alphaproteobacteria; Acetobacterales; Acetobacteraceae; Acidiphilium | 1.19 | 1.75 |
| Cyanobacteria; Cyanobacteriia; Cyanobacteriales; Chroococcidiopsaceae | 1.05 | 2.12 |
| Cyanobacteria; Cyanobacteriia; Cyanobacteriales; Nostocaceae; Nostoc PCC-73102 | 1.00 | 2.03 |
| **Top 10 ASVs in the active snow bacterial community** | **Relative ASV abundance (%)** | |
| **Taxonomic annotation** | **Snow** | **Air** |
| Cyanobacteria; Cyanobacteriia; Cyanobacteriales; Chroococcidiopsaceae | 5.09 | 2.67 |
| Cyanobacteria; Cyanobacteriia; Cyanobacteriales; Phormidiaceae; Tychonema CCAP 1459-11B | 3.16 | 6.56 |
| Proteobacteria; Alphaproteobacteria; Rhizobiales; Rhizobiaceae; Allorhizobium-Neorhizobium-Pararhizobium-Rhizobium | 2.41 | 0.15 |
| Cyanobacteria; Cyanobacteriia; Cyanobacteriales; Chroococcidiopsaceae | 2.12 | 1.05 |
| Cyanobacteria; Cyanobacteriia; Cyanobacteriales; Nostocaceae; Nostoc PCC-73102 | 2.03 | 1.00 |
| Proteobacteria; Alphaproteobacteria; Acetobacterales; Acetobacteraceae; Acidiphilium | 1.75 | 1.19 |
| Proteobacteria; Alphaproteobacteria; Acetobacterales; Acetobacteraceae; Acidiphilium | 1.62 | 1.97 |
| Cyanobacteria; Cyanobacteriia; Cyanobacteriales; Chroococcidiopsaceae; Aliterella | 1.55 | 1.45 |
| Cyanobacteria; Cyanobacteriia; Cyanobacteriales; Chroococcidiopsaceae | 1.13 | 0.33 |
| Proteobacteria; Alphaproteobacteria; Sphingomonadales; Sphingomonadaceae; Sphingomonas | 0.89 | 0.72 |

**Table S.3** List of all samples, collection methods, sites, sampling times, volumes of air collected and the air flow. Further, if the sample was sequenced and accession numbers for the DNA and cDNA libraries

| **Sample ID** | **Method** | **Site** | **Start date** | **Start time** | **End Date** | **End time** | **Duration [h]** | **Volume of air [m3]** | **Air flow [L/min]** | **Sequenced** | **Accession (DNA)** | **Accession (cDNA)** |  |
| --- | --- | --- | --- | --- | --- | --- | --- | --- | --- | --- | --- | --- | --- |
| StN-RNA-1-DNA | Impinger | 1 | 14/04/2015 | 11.15 | 14/04/2015 | 16.15 | 5.5 | 355.4 | 17.95 | X | SAMN28797772 | SAMN28797773 | |
| StN-RNA-2-DNA | Impinger | 2 | 16/04/2015 | 12.25 | 16/04/2015 | 17.25 | 5.25 | 339.2 | 17.95 | X | SAMN28797770 | SAMN28797771 | |
| StN-RNA-3-DNA | Impinger | 2 | 17/04/2015 | 16.00 | 17/04/2015 | 21.00 | 5.17 | 334.1 | 17.95 | X | SAMN28797767 | SAMN28797768 | |
| StN-RNA-4-DNA | Impinger | 1 | 18/04/2015 | 10.50 | 18/04/2015 | 15.50 | 5 | 323.1 | 17.95 | X | SAMN28797765 | SAMN28797766 | |
| StN-RNA-5-DNA | Impinger | 1 | 20/04/2015 | 08.35 | 20/04/2015 | 13.35 | 5 | 323.1 | 17.95 | X | SAMN28797761 | SAMN28797762 | |
| StN-RNA-6-DNA | Impinger | 3 | 21/04/2015 | 20.00 | 22/04/2015 | 00.10 | 4.17 | 269.4 | 17.94 | X | SAMN28797758 | SAMN28797759 | |
| StN-RNA-7-DNA | Impinger | 3 | 22/04/2015 | 14.10 | 22/04/2015 | 18.05 | 2.25 | 145.4 | 17.95 | X | SAMN28797756 | SAMN28797757 | |
| StN-RNA-8-DNA | Impinger | 3 | 23/04/2015 | 12.30 | 23/04/2015 | 17.20 | 4.83 | 312.1 | 17.95 |  | NA | NA | |
| StN-RNA-9-DNA | Impinger | 3 | 24/04/2015 | 14.55 | 24/04/2015 | 19.55 | 5 | 323.1 | 17.95 |  | NA | NA | |
| StN-RNA-10-DNA | Impinger | 3 | 25/04/2015 | 08.10 | 25/04/2015 | 13.05 | 4.92 | 317.9 | 17.95 |  | NA | NA | |
| StN-RNA-11-DNA | Impinger | 3 | 27/04/2015 | 11.10 | 27/04/2015 | 16.00 | 4.5 | 290.8 | 17.95 |  | NA | NA | |
| StN-RNA-12-DNA | Impinger | 3 | 30/04/2015 | 09.15 | 30/04/2015 | 14.15 | 5 | 323.1 | 17.95 |  | NA | NA | |
| StN-RNA-13-DNA | Impinger | 3 | 30/04/2015 | 14.20 | 30/04/2015 | 19.20 | 5 | 323.1 | 17.95 | X | SAMN28797774 | SAMN28797775 | |
| StN-RNA-14-DNA | Impinger | 3 | 30/04/2015 | 19.25 | 01/05/2015 | 00.25 | 5 | 323.1 | 17.95 |  | NA | NA | |
| StN-RNA-15-DNA | Impinger | 3 | 06/05/2015 | 16.40 | 06/05/2015 | 21.40 | 5 | 323.1 | 17.95 |  | NA | NA | |
| StN-RNA-16-DNA | Impinger | 3 | 08/05/2015 | 12.40 | 08/05/2015 | 19.20 | 6.67 | 431.0 | 17.95 |  | NA | NA | |
| StN-RNA-17-DNA | Impinger | 3 | 12/05/2015 | 17.15 | 12/05/2015 | 22.30 | 5 | 323.1 | 17.95 |  | NA | NA | |
| StN-RNA-18-DNA | Impinger | 3 | 14/05/2015 | 14.45 | 14/05/2015 | 20.00 | 5.25 | 339.2 | 17.95 |  | NA | NA | |
| R-16sum-Sample 1 | Filter | Flygers hut | 15/08/2016 | 12.10 | 16/08/2016 | 20.35 | 32.42 | 30.73 | 0.26 | X | SAMN28797743 | NA | |
| R-16sum-Sample 2 | Filter | Flygers hut | 17/08/2016 | 10.55 | 18/08/2016 | 10.15 | 23.33 | 22.12 | 0.26 | X | SAMN28797742 | NA | |
| R-16sum-Sample 3 | Filter | Flygers hut | 19/08/2016 | 20.45 | 21/08/2016 | 20.40 | 47.92 | 45.42 | 0.26 | X | SAMN28797741 | NA | |
| R-16sum-Sample 4 | Filter | Flygers hut | 22/08/2016 | 00.30 | 23/08/2016 | 10.35 | 34.08 | 32.31 | 0.26 | X | SAMN28797740 | NA | |
| R-16sum-Sample 5 | Filter | Flygers hut | 23/08/2016 | 20.05 | 25/08/2016 | 10.15 | 38.17 | 36.18 | 0.26 | X | SAMN28797739 | NA | |
| R-16sum-Sample 6 | Filter | Flygers hut | 25/08/2016 | 15.20 | 26/08/2016 | 21.20 | 30.00 | 28.44 | 0.26 | X | SAMN28797738 | NA | |
| R-16sum-Sample 7 | Filter | Flygers hut | 27/08/2016 | 14.25 | 30/08/2016 | 17.30 | 75.08 | 71.18 | 0.26 | X | SAMN28797737 | NA | |

**Table S.4** List of all snow samples, sampling sites, dates and volumes of snow collected. Further, if the sample was sequenced and accession numbers for the DNA and cDNA libraries

| **Sample ID** | **Site** | **Collection date** | **Volume of snowmelt [mL]** | **Sequenced** | **Accession (DNA)** | **Accession (cDNA)** |
| --- | --- | --- | --- | --- | --- | --- |
| StN-S-SID-1 | 1 | 28/04/2015 | 1305 |  | NA | NA |
| StN-S-SID-2A | 2 | 28/04/2015 | 1840 | X | NA | SAMN28797755 |
| StN-S-SID-2B | 2 | 28/04/2015 | 2205 |  | NA | NA |
| StN-S-SID-3 | 3 | 28/04/2015 | 2500 | X | NA | SAMN28797754 |
| StN-S-SID-4 | 4 | 28/04/2015 | 2890 | X | SAMN28797752 | SAMN28797753 |
| StN-SID-5A | 5 | 28/04/2015 | 2730 | X | SAMN28797749 | SAMN28797747 |
| StN-SID-5B | 5 | 28/04/2015 | 2405 | X | NA | SAMN28797748 |
| StN-S-SID-6A | 6 | 28/04/2015 | 1830 | X | SAMN28797749 | NA |
| StN-S-SID-6B | 6 | 28/04/2015 | 1705 | X | SAMN28797750 | NA |
| StN-SofiaA | S | 27/04/2015 | 1800 | X | SAMN28797746 | NA |
| StN-SofiaB | S | 27/04/2015 | 785 |  | NA | NA |
